# Antiscatter grid use in pediatric digital tomosynthesis imaging†

**DOI:** 10.1120/jacmp.v12i4.3641

**Published:** 2011-11-15

**Authors:** Jenna M. King, Idris A. Elbakri, Martin Reed

**Affiliations:** ^1^ Division of Medical Physics CancerCare Manitoba Winnipeg Manitoba Canada; ^2^ Department of Radiology Winnipeg Children's Hospital Winnipeg Manitoba Canada

**Keywords:** digital tomosynthesis, digital radiography, pediatrics, antiscatter grid

## Abstract

The objective of this study was to assess the effect of antiscatter grid use on tomosynthesis image quality. We performed an observer study that rated the image quality of digital tomosynthesis scout radiographs and slice images of a Leeds TO.20 contrast‐detail test object embedded in acrylic with and without a grid. We considered 10, 15, 20 and 25 cm of acrylic to represent the wide range of patient thicknesses encountered in pediatric imaging. We also acquired and rated images without a grid at an increased patient dose. The readers counted the total number of visible details in each image as a measure of relative image quality. We observed that the antiscatter grid improves tomosynthesis image quality compared to the grid‐out case, which received image quality scores similar to grid‐in radiography. Our results suggest that, in order to achieve the best image quality in exchange for the increase in patient dose, it may often be appropriate to include an antiscatter grid for pediatric tomosynthesis imaging, particularly if the patient thickness is greater than 10 cm.

PACS number: 87.57.‐s

## I. INTRODUCTION

The volumetric information and high diagnostic quality of computed tomography (CT) have led to a continuous increase in the popularity of CT in diagnostic imaging. A recent study of diagnostic imaging trends observed that the frequency of CT use is increasing at a rate of 10% per year in the United States, with rapid increases in frequency occurring in the pediatric population.^(^ ^1^ ^)^ Because CT is a relatively high‐dose imaging modality, its increasing popularity as a diagnostic tool in pediatrics is a cause for concern because the small size, rapidly dividing tissues, and long life expectancy of children at the time of exposure make them more sensitive to the damaging effects of ionizing radiation than adults.^(^ ^2^ ^)^ As such, it is important for us to continuously strive to keep patient doses as low as reasonably achievable (ALARA). Two possible ways to achieve this goal are through the development and assessment of alternative, lower dose imaging modalities or through the optimization of existing techniques.^(^ ^1^ ^,^ ^3^ ^–^ ^5^ ^)^

Digital tomosynthesis (DT) is an example of a possible alternative to CT in some situations. In DT, tomographic slice images are reconstructed parallel to the detector plane from a series of discrete projection radiographs that are collected while the X‐ray tube sweeps through a limited angle above the detector.^(^ ^6^ ^)^ Several recent studies have shown that the patient doses from DT can be significantly lower than CT for a number of diagnostic imaging applications.^(^ ^7^ ^–^ ^11^ ^)^ Many recent studies into DT image quality have focused on chest and breast imaging in adults.^(^ ^6^ ^)^ Because of the relatively high diagnostic image quality and significantly reduced dose compared to CT, we feel that DT imaging may be particularly well‐suited to some applications in pediatric diagnostic imaging. We have begun to assess the diagnostic image quality of DT relative to digital radiography (DR) and CT. We are particularly interested in using DT in the diagnosis and follow‐up of lumbar spondylolysis, which is a relatively common cause of lower back pain in teenagers.^(^ ^12^ ^)^

While there has been renewed interest in DT imaging over the last decade, it is still a relatively young modality with the potential for further optimization, particularly in terms of dose. The use of antiscatter grids in diagnostic imaging is one factor that affects the patient dose. Antiscatter grids are placed between the patient and detector surface. They improve the contrast of the image by preferentially absorbing scattered photons, which reduces the amount of scattered radiation that reaches the detector. Because the presence of the grid also attenuates primary photons, an increase in patient dose is generally required in order to maintain sufficient exposure to the detector. This increase in detector exposure was particularly important in screen‐film radiography in order to achieve the appropriate optical density of the film. However, grids are commonly used to remove scatter in flat‐panel digital imaging as well, as they can improve the signal‐to‐noise ratio (SNR)^(^ ^13^ ^)^ and reduce the image cupping effect that arises from the variation in scatter across the field of view.

Including a grid in DR results in a dose penalty of 2 to 5 times, primarily depending on the thickness of the patient, and we expect a similar dose penalty for DT. There have been fairly extensive investigations into the image quality benefits and dose penalties associated with grid use in digital radiography^(^ ^13^ ^,^ ^14^ ^)^ and digital mammography.^(^ ^15^ ^,^ ^16^ ^)^ Wu et al.^(^ ^17^ ^)^ investigated the effects of grid use in digital tomosynthesis of the breast, but to the best of our knowledge the same depth of investigation into grid use does not yet exist for DT, particularly in the context of general pediatric imaging.

Current vendor‐set default imaging protocols at our institution recommend that we include the grid for virtually all pediatric DT exams. As the grid is occasionally removed in DR when imaging small children in order to save dose, we would like to determine whether there is an obvious threshold patient thickness below which the image quality benefits that are provided by the grid would be outweighed by the increase in patient dose for pediatric DT. In addition, the grid‐out DT doses are significantly lower than the grid‐in values and, as DT is a digital imaging technique that is noise‐limited rather than contrast‐limited, it may be possible to improve the quality of grid‐out images by increasing the dose while still maintaining a much lower patient dose. Therefore, we may be able save patient dose without sacrificing a significant amount of image quality by performing DT without the grid for the vast majority of pediatric patients.

The purpose of this study was two‐fold: to assess the effect of the grid on the dose and diagnostic image quality of our current DT spinal imaging protocols, and to investigate any image quality and dose benefits that may be gained by increasing the tube current time (mAs) of DT imaging without the grid. This information can assist us in the development of clinical guidelines for grid use in pediatric DT imaging at our institution.

## II. MATERIALS AND METHODS

We performed an observer study that rated the diagnostic quality of DR and DT images acquired with and without the grid. We acquired all our images with a commercial X‐ray system with digital tomosynthesis capabilities (Definium 8000, GE Healthcare, Waukesha, WI). For this image quality assessment, we used a Leeds TO.20 X‐ray contrast‐detail test object embedded in acrylic slabs to simulate the scatter and attenuation properties of different patient thicknesses. The test object contains 144 circular details of varying contrast (0.014 to 0.924 for a 75 kV X‐ray beam with 1.5 mm of copper filtration) and diameters (between 0.25 and 11.1 mm).

### A. Image acquisition

We performed our DT imaging using a vendor‐defined lateral view spinal imaging protocol with a source‐to‐image distance (SID) of 100 cm. The DT image acquisition sequence acquires an initializing scout radiograph, which is essentially a lateral view DR image, and then acquires 60 discrete low‐dose projection radiographs as the X‐ray tube sweeps through a 30° angle above the table. The final processed tomosynthesis slice images are reconstructed from the 60 projections using a generalized filtered back projection algorithm with a 4 mm slice interval.

Initially, the TO.20 test object was placed on top of 10 cm of acrylic centered in the X‐ray field of view and the antiscatter grid (100 cm focal distance, 70 lp/mm, 12:1 grid ratio) was included, per the protocol recommendation. The scout image was acquired at 70 kV with a tube current time of 2.81 mAs. Automatic exposure control (AEC) was used to establish the exposure time. The 60 tomosynthesis projection images were also acquired at 70 kV with the grid in place. The mAs was held constant for each projection, and the value was calculated automatically from the scout image mAs using a dose ratio of 12 (i.e., the total mAs of the 60 sweep images was limited to 12 times the scout image value).

We then performed a DT acquisition (scout and sweep) using AEC without the grid, as well as an additional DT sweep acquisition without the grid where we manually increased the mAs per projection to roughly double the AEC‐determined value. Therefore, with 10 cm of acrylic we acquired three DR scout images and tomosynthesis sweeps.

**Figure 1:**
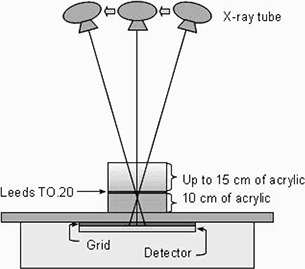
Schematic diagram of phantom setup.

We repeated this process with acrylic thicknesses of 15, 20, and 25 cm, where the additional acrylic was placed on top of the TO.20 test object and initial 10 cm acrylic slab (Fig. 1) in order to keep the distance between the test object and the point of rotation of the X‐ray tube constant in all cases. The thicknesses were chosen to represent the wide range of lateral trunk thicknesses encountered in a pediatric population.^(^ ^18^ ^)^ As we increased the total acrylic thickness, we also increased the tube potential (kV) to mimic current clinical imaging practice. We used 75 kV for 15 cm of acrylic and 80 kV for both the 20 and 25 cm cases.

As a secondary investigation, we also acquired grid‐in and grid‐out scout and tomosynthesis images of the test object with 18 cm of acrylic (i.e., a typical pediatric skull thickness^(^ ^18^ ^)^) using the vendor‐set posterior–anterior (PA) facial bone imaging protocol, which collects 60 projection images over a 40° sweep angle and uses a dose ratio of 10. The facial bone images were acquired using AEC at 75 kV.

### B. Image analysis

**Figure 2:**
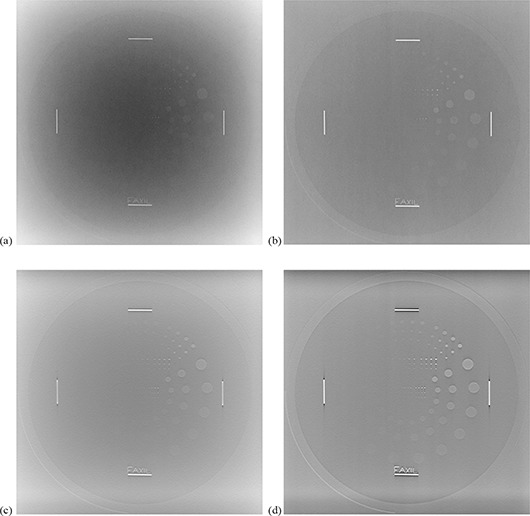
Images of the Leeds TO.20 test object with 15 cm of acrylic scatter material: (a) DR image with the grid out, (b) DR image with the grid in, (c) DT slice through the test object with the grid out, (d) DT slice through the test object with the grid in.

Four readers, two pediatric radiologists and two medical physicists, acted as readers in this study. The size and location of the contrast details were provided to the readers, and all image rating was performed on a 2 megapixel Dell 2405 FPW 24” flat panel monitor. For each imaging condition considered, each reader scored the visibility of each contrast detail on both the DR scout image and the tomosynthesis slice image with the best visibility of the Leeds test object (Fig. 2). Each of the outer, larger half of the details was rated as 1, 0.5 or 0, signifying the details were clearly seen (well‐defined boarders), partially seen, or not seen, respectively. Due to its small size, the center half of the details was rated as seen or unseen (0 or 1) only.

The readers began the study by rating three training images of the Leeds test object under different imaging conditions to familiarize themselves with the scoring system. The scores from the training images were not included in the final analysis. Once trained, the readers were presented with the study images in random order over six sessions. No time limit was imposed on the sessions, which averaged 30 minutes in length, and the images did not contain any identifying information. During the rating, the readers were free to adjust the window and level settings to their preference.

For each reader, we assigned a total image quality score to each DR and DT image by summing the scores of the individual contrast details. Because we acquired our images under different exposure conditions from those where the detail contrasts are defined, we used the total number of visible details as a measure of the relative diagnostic image quality between images rather than absolute threshold contrast‐detail detectability. To assess the effect of grid use on patient dose, we used the mAs values in the image DICOM headers to calculate the Bucky Factor (i.e., the ratio of mAs with and without the grid) for both the scout radiographs and the tomosynthesis sweeps.

### C. Signal‐difference‐to‐noise‐ratio (SDNR)

**Figure 3:**
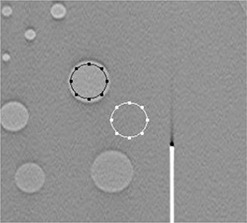
Contrast detail (black circle) and background region (white circle) for SDNR calculation.

To compare our subjective image scores with a more quantitative measurement, a single SDNR measurement was made for the largest, highest contrast detail in each rated image. The SDNR was calculated according to the following formula:

(1) $$SDNR=\frac{S_{cd}-S_{background}}{\sqrt{\sigma_{cd}^{2}+\sigma_{background}^{2}}}$$

where $S_{\mathrm{cd}}$ and $S_{\mathrm{background}}$ are the mean pixel values of the contrast detail and background regions, and $\sigma_{\mathrm{cd}}$ and $\sigma_{\mathrm{background}}$ are the standard deviation of the pixel values in the detail and background regions, respectively, as shown in Fig. 3.

### D. Statistics

Using the total scores from all readers, we assessed the internal reliability of our image rating system by calculating a Cronbach's Alpha. We also assessed inter‐ and intrareader reliability by calculating a two‐way, mixed effects intraclass correlation coefficient and a correlation coefficient matrix. All reliability statistics were calculated using SPSS statistical computing software (SPSS Inc., Chicago, IL). We compared the average scores between cases using independent t‐tests in Excel (Microsoft Inc., Redmond, WA), with two‐tailed p‐values of less than 0.05 considered statistically significant.

## III. RESULTS

### A. Reliability statistics

Our rating system showed a high level of internal reliability with a Cronbach's Alpha of 0.988. Inter‐reader correlation coefficients ranged from 0.932 to 0.982, and the intraclass correlation coefficient was 0.955 with a 95% confidence interval of 0.934 to 0.971, indicating agreement between the four readers.

### B. AEC image quality scores

**Figure 4:**
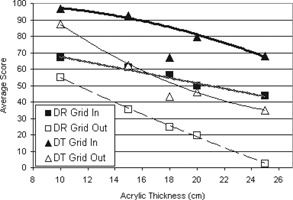
Mean image quality scores for DR and DT, using AEC with and without the grid. The trend lines shown are second order polynomial lines of best fit.

When the images were acquired using AEC, there was a statistically significant improvement in the image quality score for both DR and DT at all acrylic thicknesses (*p* from < 0.001 to 0.044) when the grid was included, with the exception of DT at 10 cm (Fig. 4). In that case, the difference between the grid‐in and grid‐out score was not statistically significant (p=0.992). Despite the inherently higher dose of DT, the grid‐out tomosynthesis scores were not statistically different from those for DR with the grid, except for the 25 cm case, where the DR image score was higher than the tomosynthesis image score (p=0.004).

### C. Dose assessment

Similar to DR, we observe significant increases in mAs, and therefore patient dose, when the grid is included in DT imaging (Table 1). In fact, the increase in dose associated with the grid is greater with DT than DR for the thicker phantoms. This is most likely due to the limitations on the possible mAs setting for each projection image due to the dose ratio and X‐ray system design.

**Table 1:** Effect of grid on total mAs in lateral lumbar spine imaging (AEC).

	*Digital Radiography (DR)*			*Digital Tomosynthesis (DT)*		
*Acrylic Thickness (cm)*	*Grid IN*	*Grid OUT*	*Bucky Factor*	*Grid IN*	*Grid OUT*	*Bucky Factor*
10	2.8	1.0	2.8	30	15	2.0
15	5.4	1.5	3.6	60	15	4.0
20	10.2	2.2	4.6	120	24	5.0
25	27.7	5.1	5.4	300	48	6.3

### D. Increasing the mAs per projection for DT without the grid

**Figure 5:**
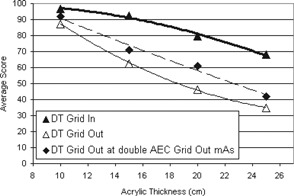
Mean image quality scores for DT imaging with and without the grid and when the grid‐out mAs setting is manually doubled. The trend lines shown are second order polynomial lines of best fit.

From Fig. 5, we can see that manually doubling the grid‐out mAs suggests an increase in image quality score, although this increase was only statistically significant for the 20 cm case (p=0.026). In that case, the mAs per projection had to be slightly more than doubled due to the limited selection of mAs values available on the machine (Renard steps). In all cases, the doubled grid‐out mAs values, and therefore patient doses, are less than half of the grid‐in values. However, the observed image quality is still lower than the grid‐in case (*p* between 0.003 and 0.02) for all acrylic thicknesses except 10 cm, where there are no significant differences in score between any of the cases considered.

### E. SDNR

**Figure 6:**
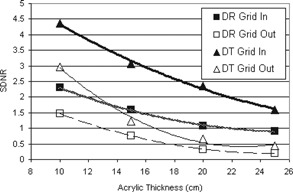
Signal‐difference‐to‐noise ratio versus acrylic thickness for DR and DT imaging with and without the grid using AEC. The trend lines shown are second order polynomial lines of best fit.

As Fig. 6 shows, the SDNR calculations have similar trends as the qualitative image quality scores. Grid‐in DT has the highest SDNR, as well as the highest image quality scores, while DR without the grid results in both the lowest SDNR and image quality scores. In all cases, both the SDNR and image quality score decrease with increasing acrylic thickness, as scatter contribution also increases.

### F. Facial bone protocol

**Figure 7:**
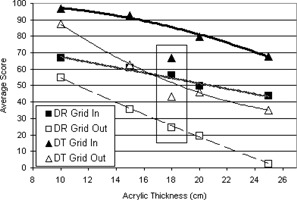
Mean image quality scores for DR and DT, using AEC with and without the grid, including the results of the facial bone protocol (18 cm). The trend lines are second order polynomial lines of best fit through the spinal imaging protocol data only.

The image quality scores for facial bone DR imaging are what we would have expected for 18 cm of acrylic based on the trends we observed for the DR spinal imaging scout radiographs (Fig. 7). However, the DT facial bone sweep score is lower than the trend we observed for the spinal imaging DT sweeps, both with and without the grid. These lower scores are likely the result of the lower projection density and dose ratio of the facial bone imaging protocol compared to the spinal imaging protocol. However, the wide sweep angle of the facial bone protocol allows for a thinner slice profile, which is desirable when visualizing the fine details present in facial bone anatomy.

## IV. DISCUSSION

The results of our study suggest that tomosynthesis imaging should be performed with a grid unless the patient is very thin, roughly 10 cm or less in thickness. This result is consistent with common guidelines for digital radiography. When investigating the use of grids and air gaps as scatter reduction techniques in DR, Neitzel^(^ ^14^ ^)^ concluded that grid use can degrade image quality under low‐scatter conditions because of the attenuation of primary photons, but it is appropriate to use the grid for digital radiography of the torso, where there is a large amount of scatter. Similarly, our study suggests that we should use antiscatter grids for pediatric lateral view digital tomosynthesis spinal imaging, where we observe some of the widest patient thicknesses and highest scatter conditions encountered in pediatric imaging. In a 2007 study, Raissaki^(^ ^5^ ^)^ recommends that an antiscatter grid be used in radiography for patient thicknesses greater than 10–13 cm, and when investigating the usefulness of an antiscatter grid for digital mammography, Veldkamp et al.^(^ ^16^ ^)^ concluded that the grid could be removed for thicknesses up to 7 cm, which is also consistent with our observations.

Wu et al.^(^ ^17^ ^)^ found that the presence of scatter significantly degraded the image quality (as measured by signal‐difference‐to‐noise ratio) when no antiscatter grid was used for tomosynthesis imaging of the breast. They also measured similar image quality between tomosynthesis imaging without the grid and radiographic imaging with a grid; however, they were considering equivalent exposure levels, while our AEC imaging protocols resulted in grid‐out tomosynthesis doses that were higher than grid‐in radiography by approximately 2–5 times. We also observed a decrease in the SDNR when no antiscatter grid was used for DT. A more extensive SDNR investigation could be performed in future studies, but in this case the phantom contained many small details that could not be seen on several images. The largest, highest contrast detail could be identified in every image and its SDNR allowed us to provide a convenient, quantitative comparison between images which the results showed to correlate well with the subjective rating of images.

Doubling the patient dose of DT without the grid improved the image quality score, although the improvements were generally not statistically significant, likely due to the small scale of this study. However, the patient dose is still less than half of the grid‐in tomosynthesis value when the grid‐out mAs is doubled. We had originally considered the case of grid‐out DT at the same mAs as grid‐in DT, but we ultimately decided against including it in our study. Our reasoning for this was that without the grid to remove scatter, the images would at best be similar in quality to the grid‐in images. We felt that if we were going to be using the same mAs (and therefore delivering the same patient dose) as the grid‐in case, there would be no motivation to remove the grid. Therefore, we chose to only rate the grid‐out tomosynthesis images at the AEC‐determined mAs and two times that value so that there would always be a reduction in patient dose associated with grid‐out imaging. With some idea of the relative image quality for these lower‐dose cases, we may be able to identify situations where grid‐out tomosynthesis would provide acceptable images for the diagnostic task of interest without having to subject the patient to the full grid‐in tomosynthesis dose. Whether or not the grid is used, DT doses are significantly lower than CT doses.

It is difficult, if not impossible, to define a minimum score that constitutes acceptable diagnostic image quality, as pediatric imaging can be performed for a wide variety of reasons, each with unique objectives. We should note that grid‐out DR also resulted in low scores, even for the thinnest patient case considered, but is still performed when the quality of the resulting image will be sufficient for the intended purpose. The same may be true of tomosynthesis, which may provide additional diagnostic benefits over radiography that we could not observe with this study. We only considered a single tomosynthesis slice through a test object that did not include any over‐ or underlying structures, and one of the proposed strengths of tomosynthesis is its ability to reduce the interference of anatomical structural noise. In each tomosynthesis slice, the out‐of‐plane structures are blurred, thus improving the contrast and visibility of the in‐plane structures compared to a two‐dimensional projection radiograph. Wu et al.^(^ ^17^ ^)^ found that the benefits of breast DT became more apparent when realistic anatomic noise was included in the simulation. On a related note, the depth information afforded by the volumetric nature of a tomosynthesis image set can also provide valuable diagnostic information that would not be observed in this investigation.

The need for an antiscatter grid may be reduced through the use of alternative scatter correction techniques. For example, Siewerdsen et al.^(^ ^19^ ^)^ proposed an X‐ray scatter correction algorithm for flat‐panel, cone‐beam CT that models the scatter fluence of each projection image. They found that applying the correction was roughly equal to the performance of a heavy antiscatter grid, and proposed that their method is not limited to CBCT but could be applied to other flat‐panel digital imaging techniques like DT. Similarly, Tromans et al.^(^ ^20^ ^)^ investigated a software scatter correction for digital mammography, and found that the performance of the scatter correction was roughly equivalent to that of the grid, and the images maintained a higher signal‐to‐noise ratio. Such digital scatter corrections during image processing and reconstruction may provide a method to keep patient dose low, while maintaining the diagnostic quality of DT performed with the grid.

## V. CONCLUSIONS

In general, including the grid improves the average image quality score for both digital tomosynthesis and digital radiography. In order to achieve the best possible image quality in exchange for the increase in patient dose inherent to DT imaging, our results suggest that we may want to include the grid for most pediatric spinal imaging, particularly when the patient thickness is greater than 10 cm. Depending on the purpose of the imaging, it may be possible to obtain adequate tomosynthesis images without the grid in order to save dose. Further investigation into the potential diagnostic advantages of digital tomosynthesis is warranted.

## ACKNOWLEDGMENTS

Research funding for this study was provided by GE Healthcare (Waukesha, WI) and the Winnipeg Health Sciences Centre (Winnipeg, MB). This research was made possible by the helpful cooperation of Dr. Jens Wrogemann and the pediatric radiology staff at our institution.
